# Emilia Huerta-Sanchez

**DOI:** 10.1093/gbe/evac178

**Published:** 2023-01-06

**Authors:** Adri K Grow

**Affiliations:** Department of Biological Sciences, Smith College, Northampton, MA 01063, USA

**Figure:**
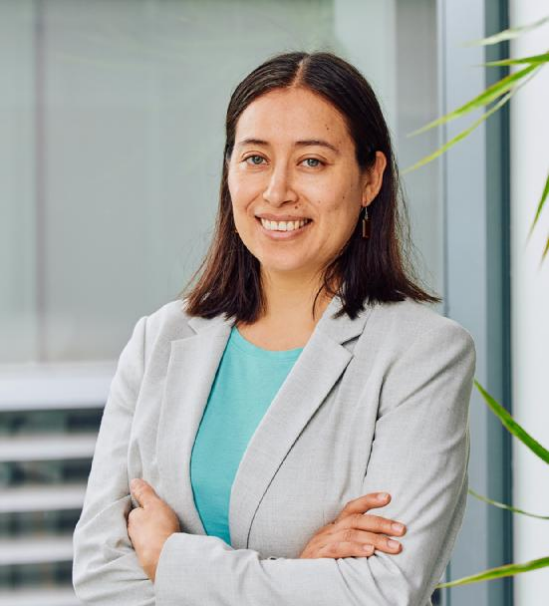


Dr. Emilia Huerta-Sanchez, Associate Professor of Ecology, Evolution, and Organismal Biology, Brown University.

We continue our biography section, featuring Dr. Emilia Huerta-Sanchez. The following is based on a September 2022 interview with Emilia.

## How did You Become a Scientist?

Growing up in Mexico and later moving at the age of 11 to Los Angeles, Emilia's primary motivation in school was to get a good job so that she could provide for her family. A career in scientific research was not where she saw herself ending up. Emilia had every intention of studying economics and French in college, but during her first year at Mills College in Oakland, California, she met a math major who sparked the pivotal change that shaped her future career. This student was talking about her summer research experience for undergraduates (REUs) program in Puerto Rico and Emilia was amazed that such a program existed that covers all expenses and allows you to see different parts of the country. Having taken many math classes, Emilia decided that she would become a math major so she could apply to the same REU program, but would switch back to something else after the summer. Emilia was accepted into the program and not only enjoyed the work she was doing but was also exposed to new information about graduate school and the funding available for different PhD programs. That following summer, Emilia was accepted into an REU program at Cornell University. This time instead of a sole focus on math, the project was based in mathematical and theoretical population biology. This was the type of science and field that Emilia saw in her future.

After having a really enjoyable experience in the summer program, Emilia liked the environment at Cornell so much that she attended Cornell for her graduate studies. Emilia obtained a Master's and PhD in Applied Mathematics from Cornell University. The applied mathematics program was quite flexible and Emilia was able to choose co-advisors from two different departments for her PhD. One of her PhD co-advisors was Dr. Richard Durrett, who focuses on probability, stochastic processes (one of Emilia's favorite classes she took in graduate school), and population genetics. Emilia's other PhD co-advisor was Dr. Carlos Bustamante, whose focus is on statistical inference and population genetics. After getting her PhD, Emilia was an NSF postdoctoral research fellow in the Departments of Statistics and Integrative Biology at the University of California, Berkeley working in Dr. Rasmus Nielsen's laboratory. This was an exciting time for Emilia as next-generation sequencing was an emerging tool along with the advent of whole-genome sequencing. Emilia took advantage of the amount of data that were being produced and had the opportunity to work on many different projects as a postdoc. At this point, Emilia was increasingly interested in data information, genetics, and statistical analyses and focused her postdoctoral research in these areas.

Today, Emilia is an associate professor of Ecology, Evolution, and Organismal Biology and a member of the Center for Computational Molecular Biology (CCMB) at Brown University. Her research focuses on human genome evolution, population genetics, and statistics.

## Who has been Your Biggest Mentor or Influence on Your Career?

Retrospectively, Emilia says there have been many influential steps in her pathway that led her to where she is today. Emilia emphasizes that all of her mentors have been important at different stages in her career, but that mentorship doesn’t need to be a strict role. The student that introduced Emilia to the REU program had the biggest influence on her career, exposing Emilia to a completely new field that she otherwise wouldn’t have known about. What Emilia finds special about this moment is that it was a one-off conversation with another student that allowed her to find her passion rather than a specific mentor in her life. It affirmed for her that every interaction truly does have the ability to change your life. Having the ability to participate in programs like REUs can open a lot of doors or at the very least inform students of the different options available to them. Emilia has gotten a lot out of her research experiences from those that served in mentorship roles and now bumps into them at conferences as a fellow colleague.

Emilia's PhD and postdoc advisors served as excellent mentors during her training, each being influential in providing a positive and productive environment to conduct research surrounded by other scientists working on exciting projects. Emilia also stresses just how important commiserating with other graduate students was in developing a well-rounded support system.

## What are Some Challenges You’ve Faced in Your Career?

The transition from undergraduate to graduate studies was one particular challenge in Emilia's career. Emilia recalls wanting to quit after the first year of graduate school because she was struggling to do well in her classes. In Emilia's PhD program, coursework needed to be completed first before diving into research, and Emilia felt as though if she couldn’t do well in the classes, would she be able to make it through the program at all? Emilia took those feelings of doubt and channeled them into a challenge to prove to herself that she was capable of persisting through a difficult transition. Understanding that graduate classes have less explicit instruction and are more independently led was an important step to success for Emilia. Her classes required more independent study and reading than she was accustomed to as an undergraduate. One thing that made it easier was the support of her graduate cohort and the comradery of going through similar struggles together.

Aside from the courses, Emilia made the switch from a historically all-women's college in undergrad to a co-ed graduate program. This change also affected Emilia's feeling of belonging as she was often one of the very few (two to three) women in the classes she took. Emilia was confronted with hearing things like “women aren’t good at math,” which influenced the way she thought about herself in academia for some time. As an underrepresented minority, Emilia felt a lot of pressure to do well because if she didn’t, it would negatively impact anyone like her following a similar trajectory. Emilia also felt like she needed to continually prove to other people (and herself) that she had the ability to be in the program and conduct high-quality research. It took Emilia a while to come to terms with belonging in a historically male-dominated field and to feel secure, but today she is confident in her career and more easily able to ignore what people think of her.

## What do You do for Fun Outside of Science?

Emilia loves to travel, but her philosophy today in a climatically fragile world is to travel with intention. Emilia focuses on traveling to places where she has friends so that she can travel while also fostering personal relationships at the same time. Emilia enjoys running with her CCMB colleagues Dr. Sohini Ramachandran and Dr. Ritambhara Singh. Emilia finds that exercise helps her maintain a well-balanced life along with adequate sleep. She strongly recommends that everyone get at least eight hours of sleep a day!

## Your Favorite Contribution to the Literature?

Emilia has been working on high-altitude adaptations, including linking a specific gene involved in the regulation of the body's response to low-oxygen conditions found in the Tibetan population. Her paper titled “Sequencing of 50 human exomes reveals adaptation to high altitude” was published in 2010 in *Science*. As a result of this paper, Emilia was able to see just how different the Tibetan haplotype was from other populations. Emilia noted that the haplotype was so different that it most likely didn’t originate in Tibet and likely came from another population. Emilia started sifting through the genomes available in the 1000 Genomes Project but didn’t find anything similar among modern human genomes. In conversation with her fellow postdocs who worked on archaic introgression, Emilia got her hands on some archaic sequences to compare with the Tibetan sequences and found an exact match with Denisovans. Emilia never thought that archaic introgression would be a part of her research, but it led to one of her most exciting research projects and her favorite contribution to the literature published in *Nature* in 2014 titled “Altitude adaptation in Tibetans caused by introgression of Denisovan-like DNA.”

## What's Some Advice for People Entering the Field of Science?

Science is inherently fluid and Emilia says that too much pressure shouldn’t be placed on finding perfect projects to work on. Emilia likes to start with an idea and explore all the possible facets an idea can be approached from as it often leads her down unexpected paths. Emilia also states how important writing is, especially the further you get in your career. Even if you’re not focused on writing grants and manuscripts all the time, Emilia stresses that writing is one way to effectively communicate with one another and it is an essential skill to have as a scientist. Finding a strategy for writing is really beneficial, whether it be accountability groups, writing a little bit every day rather than big chunks at once, or blocking out time dedicated to writing. Emilia shares that her writing strategy is to completely disconnect from the internet so that she is forced to focus on writing. There is no option to be sidetracked by looking something up while writing that eventually leads back to sifting through your email. Emilia disconnects and sets a 30-minute timer and if she really needs to look something up she’ll write it down and go back to the writing. She also occasionally writes with a buddy so they can keep each other accountable and reach their writing goals together. The last piece of advice from Emilia is to remember that science is a long game. Emilia had young children when she started her position as a professor and felt like she was missing out on a lot of science adapting to a new work-life balance but ultimately, she allowed herself the grace and patience to set long-term goals and find the science that was most intellectually rewarding for her.

## Any Final Thoughts?

Emilia loves being a professor at Brown and loves the collaborative nature of science. Working with undergraduates, graduate students, postdocs, and other colleagues is something that brings her a lot of joy. Emilia enjoys being able to brainstorm with her students and learn through the lens of her students and collaborators. One of Emilia's favorite things about her career is being able to see the diversity of trajectories that students and colleagues have taken in their own personal journeys in science.

